# Implementation plans included in World Health Organisation guidelines

**DOI:** 10.1186/s13012-016-0440-4

**Published:** 2016-05-20

**Authors:** Zhicheng Wang, Susan L. Norris, Lisa Bero

**Affiliations:** 1 Faculty of Pharmacy, The University of Sydney, Sydney, New South Wales Australia; 2 World Health Organization, Geneva, Switzerland; 3 Charles Perkins Centre, The University of Sydney, D17, The Hub, 6th floor, Sydney, New South Wales Australia

**Keywords:** Implementation techniques, Implementation strategies, WHO, Guidelines, Guidelines Review Committee, Implementation, Clinical practice guidelines

## Abstract

**Background:**

The implementation of high-quality guidelines is essential to improve clinical practice and public health. The World Health Organisation (WHO) develops evidence-based public health and other guidelines that are used or adapted by countries around the world. Detailed implementation plans are often necessary for local policymakers to properly use the guidelines developed by WHO. This paper describes the plans for guideline implementation reported in WHO guidelines and indicates which of these plans are evidence-based.

**Methods:**

We conducted a content analysis of the implementation sections of WHO guidelines approved by the WHO guideline review committee between December 2007 and May 2015. The implementation techniques reported in each guideline were coded according to the Cochrane Collaboration’s Effective Practice and Organisation of Care (EPOC) taxonomy and classified as passive, active or policy strategies. The frequencies of implementation techniques are reported.

**Results:**

The WHO guidelines (*n* = 123) analysed mentioned implementation techniques 800 times, although most mentioned implementation techniques very briefly, if at all. Passive strategies (21 %, 167/800) and general policy strategies (62 %, 496/800) occurred most often. Evidence-based active implementation methods were generally neglected with no guideline mentioning reminders (computerised or paper) and only one mentioning a multifaceted approach. Many guidelines contained implementation sections that were identical to those used in older guidelines produced by the same WHO technical unit.

**Conclusions:**

The prevalence of passive and policy-based implementation techniques as opposed to evidence-based active techniques suggests that WHO guidelines should contain stronger guidance for implementation. This could include structured and increased detail on implementation considerations, accompanying or linked documents that provide information on what is needed to contextualise or adapt a guideline and specific options from among evidence-based implementation strategies.

## Background

The implementation of evidence-based guidelines can improve clinical and public health outcomes by helping health professionals practice in the most effective and efficient manner and policymakers design optimal programmes. The development of guidelines without adequate implementation plans may hinder the targeted audiences’ adherence to the guidelines [1]. The implementation of guidelines with effective, evidence-based techniques may ultimately lead to better outcomes for the target population [2].

The World Health Organisation (WHO) is a major contributor to global practice guidelines as many countries around the world adopt or adapt WHO guidelines. In response to criticism of its guideline quality and processes used for development [3], WHO established the Guidelines Review Committee (GRC) in 2007 to ensure and improve the quality of their guidelines. This committee meets on a monthly basis to review guideline planning proposals as well as the final version of guidelines prior to their publication. The GRC implemented standards and methods for guideline development based on evidence and implemented the Grading of Recommendations Assessment, Development and Evaluation (GRADE) approach to guideline development [4]. The WHO Handbook for guideline development (2nd edition, 2014) [5] describes the current WHO methods and standards.

Although the quality of WHO guidelines has improved steadily [6, 7], there have been no studies of the implementation techniques proposed in WHO guidelines. The WHO Handbook for Guideline Development provides guidance for managing conflicts of interest in guideline development, conducting systematic reviews, grading the strength of recommendations and guideline implementation [5]. However, in the most recent edition of the Handbook, the description of what should be in the implementation section is relatively brief [5].

This study aims to describe plans for guideline implementation contained in all WHO guidelines approved by the GRC from its inception in December 2007 to May 2015. WHO defines a guideline as “any document developed by the World Health Organization containing recommendations for clinical practice or public health policy” [5].

The effectiveness of many implementation techniques has been assessed in previous studies and reviews [8–10]. We determined whether the techniques described in WHO guidelines are based on evidence of effectiveness. We conclude with recommendations for the section on implementation in future editions of the WHO Handbook for Guideline Development [5].

## Methods

We conducted a content analysis of the implementation sections of all WHO guidelines approved by the GRC between December 2007 and May 2015. This study focused on the guidelines published after the establishment of the GRC in 2007 as guidelines were more standardised and used specific methods. These guidelines were coded according to the implementation techniques they described.

### Guideline identification and inclusion criteria

A WHO official provided a list of all WHO guidelines approved by the GRC as of May 2015 (*n* = 186). All guidelines on this list were reviewed for inclusion.

Guidelines were excluded from the study if they were

- Updates (the most recent guideline was included)
- Consolidated guidelines (i.e. one document that is contains content from multiple guidelines)
- Recommendation charts (i.e. mostly pictorial charts for field use)
- Model chapters for textbooks
- Interim policy guidance statements (the full guideline was included, not the interim statement)
- Position papers
- Toolkits or handbooks for field use

**Fig. 1:**
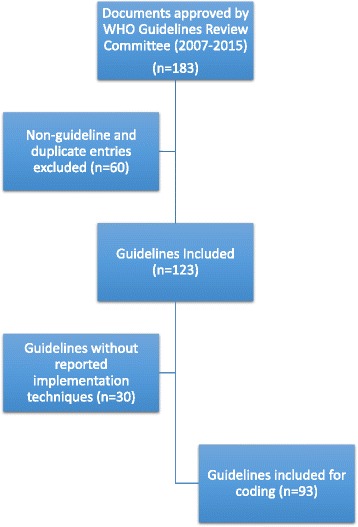
Flowchart

The documents provided by WHO were first screened according to the inclusion and exclusion criteria for this study. Guidelines which had no mention of any implementation techniques were exempt from further coding (Fig. 1). The guidelines which mentioned any implementation techniques were then coded according to the implementation techniques that they described (see Table 1).

**Table 1:** Definitions of implementation categories

Technique category	Sub-category	Definition
Education outreach	Lecture-based workshops	Continuing education workshop for health professionals that are lecture based and does not mention interaction. (keywords: workshops)
	Interactive workshops	Continuing education for health professionals in joint, interactive learning (keywords: interactive) (may/may not include evaluation component)
	Training	Teaching healthcare providers about the recommendations of the WHO guideline (keywords: Training, Training programs, Education of health professionals, continuing education) (any teaching that is not otherwise specified)
	Communication materials (posters, leaflets, flip charts, job aids)	Distribution to individuals, or groups, of educational materials to support clinical care, i.e. any intervention in which knowledge is distributed (keywords: training material, handouts, flow charts, posters, leaflets, flip charts, job aids, CD-ROM, slide presentations)^a^
	Educational follow-up/practice visits	Personal visits by a trained person to health workers in their own settings, to provide information with the aim of changing practice. (keywords: practice visits, mentoring)^a^
Information dissemination	Hard copies of guidelines	Published guidelines on paper/print (keywords: print, hard copy)
	Electronic publishing	Published guidelines electronically and/or online (keywords: website, emailing list, e-repository, elibrary)
	Research briefs	Summary of the evidence that informed the guideline, either as a separate document or supplementary material. (keywords: supplementary research material, research briefs)
	Translation of documents from English to other languages	Mentions translation
	Publications in technical and scientific journals	Publication of the guidelines and/or their development process in technical and scientific journals (keywords: journal, peer reviewed)
	Policy briefs	A written concise summary of the guideline/focused on policy implications (keywords: policy brief)
	Presentations	A speech or talk in which the guideline is shown and explained to an audience (keywords: presentation, briefing)
	Conferences	A formal meeting for discussion, launch event at a medical/scientific conference (keywords: conference)
Mass media campaigns		Wide distribution and promotion of the guideline through mass media (keywords: mass media, TV, billboards, blogs, social marketing, campaign)
Audit/feedback/peer review	Monitoring and evaluation surveys	Mention monitoring and evaluation of the implementation/uptake of guidelines (keywords: survey, register screening, monitoring, evaluation)
	Supervisory tools w/constructive feedback	Routine formal supervision visits by health staff.
	Interrupted time series clinical audits	A quality improvement process conducted in set timeframe from initial guideline implementation
	Criterion based clinical audits	A quality improvement process based on a set of criteria
	Subsequent reminders	Manual reminders to prompt health worker to follow the recommendations
	Computer-delivered reminder/clinical support systems	Computerised interventions that prompt health workers to perform an action during a consultation with a patient, for example computer decision support systems
Patient-mediated		Actively engage patients to improve their knowledge, experience, service use, health behaviour, and health status [29]
Use of local opinion leaders		The identification and use of identifiable local (country-based) opinion leaders to promote good clinical practice (keywords: opinion leaders, experts, clinician associations)^a^
Policy regional/national/local	Local consensus building/consulting stakeholders	Formal or informal local consensus processes, for example agreeing on a clinical protocol to manage a patient group, or promoting the implementation of guidelines (keywords: consensus building, stakeholders, participatory process)
	Laws, legal policies and framework	Laws, legal policies and framework set out for the implementation of the guideline (keywords: policy, law, framework)
	National standards and guidelines (adaptation)	Adapting a guideline for a local health system and setting national standards
	WHO country offices	Mention of distribution to WHO country offices
	WHO regional offices	Mention of distribution to WHO regional offices
	Government ministries/Ministry of Health	Mention of distribution to Ministry of Health
	WHO collaborating offices	Mention of distribution to WHO collaborating offices
	UN offices	Mention of distribution to UN offices/agencies
	NGOs	Mention of distribution to NGOs
	Implementing partners	Mention implementing partners, e.g. InterAgency Task Team, PEPFAR, PMTCT/Peds Technical Workgroup, Global Fund
Financial intervention	Reimbursement	Compensation paid (to someone) for health service provided; changes to reimbursement scheme
	Mention of budgets for training programmes	Mention of budgets for training programmes
	Mention of financial resources, human resources, infrastructure or equipment	Mention of financial resources, human resources, infrastructure or equipment

*WHO* World Health Organisation, *NGOs* non-government organisations, *UN* United Nations, *PEPFAR* The United States President’s Emergency Plan for AIDS Relief, *PMTCT/Peds Technical Workgroup* prevention of mother-to-child transmission (PMTCT) of HIV/paediatric technical workgroup

^a^Adapted from *Effective Practice and Organisation of Care (EPOC). EPOC Taxonomy; 2015. Available at:* *https://epoc.cochrane.org/epoc-taxonomy*

### Development of tool for categorising implementation techniques

We devised a coding tool to categorise implementation techniques mentioned in each guideline. The strategies were grouped into passive or active interventions as previous reviews found that passive implementation techniques are less effective in changing practice than active techniques [9, 11]. In contrast, numerous studies have confirmed the effectiveness of active techniques in implementing different types of guidelines [9]. Active implementation techniques include follow-up up and personal interaction with the implementers and include educational outreach, audit and feedback, reminders and use of opinion leaders [9, 11]. In contrast, passive techniques such as handouts and the dissemination of web information are limited simply to the provision of information [8, 10]. We also included a third category of policy-based techniques as WHO guidelines are often aimed at the ministries of health of United Nation Member States, as well as policymakers at the subnational and local level rather than individual health practitioners. Policy-based techniques include recommendations to the local government to develop policies or governance arrangements that optimise uptake of the guideline. These techniques could involve consulting stakeholders before implementing a recommendation, adapting the guideline to local settings and financial incentives for the target audience to increase guideline adherence. These categories were not mutually exclusive and the implementation section of a guideline could contain active, passive and policy techniques.

Our categorization scheme and coding tool were primarily based on the Cochrane Collaboration’s Effective Practice and Organisation of Care (EPOC) taxonomy [12], which provides a list of guideline implementation techniques. We also conducted a systematic review of evaluations of guideline implementation in low-income countries [13] to supplement the EPOC taxonomy and identified 14 evaluations conducted in low-income countries. This review identified effective active implementation techniques including.

1. Audit and feedback—where the target population’s guideline adherence is audited and feedback is provided [14].
2. Educational outreach—where education about the guideline is provided (through a variety of mediums) to the target population [2].
3. Reminders—where the target population is given reminders (through a variety of mediums, e.g. electronic or paper) to use the guidelines in their everyday practice [15, 16].
4. Multifaceted approaches—where a variety of implementation techniques are used to implement the guideline to the target population [16, 17].

All techniques that were studied specifically in low-income countries were already in the EPOC taxonomy.

To test and improve the reliability of our coding tool, we generated a random sample of 20 % (*n* = 19) of the guidelines which mentioned implementation techniques for coding by two reviewers. Disagreements were resolved by consensus discussion and any disagreements that could not be resolved were reviewed by a third coder. Clarifications of the definitions of the coding categories were then added to the tool as indicated (Table 1). The EPOC taxonomy, the basis for our tool, was aimed at implementation techniques that “bring about changes in healthcare organizations, the behaviour of healthcare professionals or the use of health services by healthcare recipients” [12], while WHO guidelines target a wider public health audience, including, for example public health departments, governments and NGOs. Many categories such as “communication materials” were collapsed and rearranged as sub-categories under broader categories such as “educational outreach” to accommodate the scope of WHO guidelines (Table 1). All of the categories also had keywords added to their definitions to clarify what the guidelines had to mention for an implementation technique to be coded as present.

### Data extraction

After the coding tool was revised and clarified, another 20 % of the guidelines (*n* = 19) were randomly selected to be double coded. The quality of the coding tool was improved to achieve a percentage agreement of 89 % (calculated as the number of agreement cells divided by the total number of coded cells). The first reviewer then coded the remainder of the sample with the final coding tool.

### Data analysis

The results were recorded in Microsoft Excel 2013. We conducted a descriptive statistical analysis to calculate relative frequencies of the techniques. Data are presented in cluster frequency graphs.

## Results

**Fig. 2:**
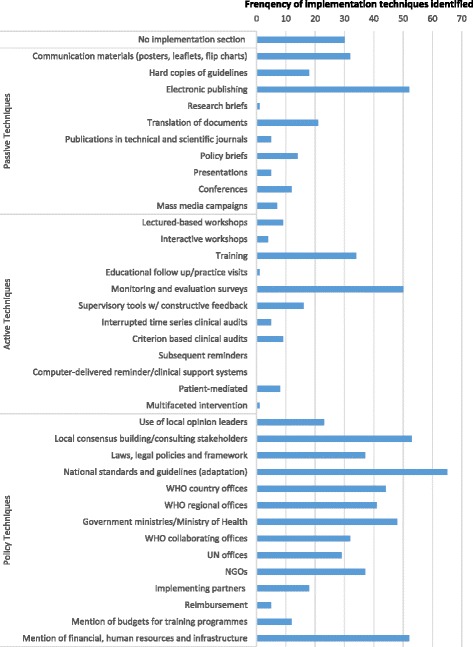
Frequency of implementation techniques in 123 WHO guidelines

Of the 186 documents provided by WHO, 123 met the inclusion criteria for our study (see Fig. 1), 93 of which included specific guidance for implementation of the recommendations. The implementation sections, if any, were often brief, but even so, guidelines were included if they contained any of the keywords defined in the coding tool. The 93 guidelines mentioned implementation techniques a total of 800 times as multiple techniques could be mentioned in a guideline. The frequency of references to each implementation technique is presented in Fig. 2. The most prevalent implementation techniques included adaptation of national standards and guidelines (*n* = 65), local consensus building/consulting stakeholders (*n* = 53) and electronic publishing of the guideline (*n* = 52). The vague and non-descriptive nature of implementation plans was not associated with any particular category of implementation techniques. Most implementation techniques in the guidelines were described with a single word or phrase, lacking clear definition for what was needed to achieve implementation.

The most prevalent techniques were policy (62 %, 496/800) and passive (21 %, 167/800) techniques (Fig. 2). These categories were not mutually exclusive and policy, and passive strategies were often recommended in combination. The most common passive techniques involved electronic publication of the guidelines (*n* = 53) and distribution of guideline summaries as poster, leaflets or in other documents (*n* = 32). The most common policy techniques were adaption of the WHO guideline to national guidelines or standards (*n* = 65) and building local consensus to support use of the guideline (*n* = 53). Frequently, little detail was provided to guide how the policy changes should be achieved, for example, “the guideline is recommended to be adapted to the context of each Member State by the local health department” [18].

In addition, when WHO offices were mentioned, detailed guidance in how to contextualize or adapt the guidelines before implementation was typically not offered in the implementation plans. One exception was the guideline “Optimizing health worker roles to improve access to key maternal and newborn health interventions through task shifting” [19]. This guideline contained a section and detailed workbook intended to help policymakers contextualise the guideline recommendations using an eight-step process: (1) clarify the problem, (2) frame the options, (3) identify implementation considerations, (4) consider the broader health system context, (5) consider the broader political system context, (6) refine the statement of the problem, options and implementation considerations in light of health system and political system factors, (7) anticipate monitoring an evaluation needs and (8) make national policy recommendations or decisions.

The most neglected of the effective implementation techniques were active techniques (Fig. 2). The techniques ‘reminders’ and ‘multifaceted approach’, in particular, had extremely limited representation in WHO guidelines. No guideline mentioned reminders (computerised or paper), and there was only one brief mention of a multifaceted approach. Of the relatively few active implementation techniques mentioned, training (*n* = 34) and monitoring and evaluation surveys (*n* = 50) were the two most frequently observed (Fig. 2.). However, when these categories were mentioned, only non-specific descriptions of the techniques were provided. Most references to training were a single word or phrase, without a clear definition of what the training should entail. For example, Personal Protective Equipment in the Context of Filovirus Disease Outbreak Response—Rapid advice guideline (2014) notes that: ‘Implementing these recommendations will require training that is suitable for different categories of health workers (including supervisors)’ [20]; however, there is no reference to where these training materials can be found or how the training should be conducted.

Another finding in this study was that a number of technical units at WHO appear to have a standardised format for their implementation section. For example, the following implementation recommendation appears in *Guideline: Intermittent iron and folic acid supplementation in menstruating women (2011)* and is representative of the nutrition health technical unit’s approach:

A plan for monitoring and evaluation with appropriate indicators is encouraged at all stages. The impact of this guideline can be evaluated within countries (i.e. monitoring and evaluation of the programmes implemented at national or regional scale) and across countries (i.e. the adoption and adaptation of the guideline globally) [21].

The identical quotation appears in the maternal health technical unit’s guideline *Optimal serum and red blood cell folate concentrations in women of reproductive age for prevention of neural tube defects (2015).*

This phenomenon repeatedly presented itself in different forms throughout our review, suggesting that the recycling of entire implementation sections is common practice across groups developing guidelines at WHO.

The implementation section of guidelines improved in detail and consistency from 2012, which coincided with the development of the first version of the *WHO Handbook for Guideline Development* [5]. Before the publication of the 2012 handbook, guidance on implementation was generally scattered throughout the guideline. The Handbook now requires an implementation section, and more recent guidelines have such a section, however brief, at the end of the document.

## Discussion

Examination of the implementation techniques suggested in all guidelines approved by the WHO Guidelines Review Committee since 2007 shows a general lack of emphasis on implementation. The implementation sections are often brief, repetitive across different guidelines, and do not recommend implementation techniques for which there is evidence of effectiveness.

WHO guidelines generally favour passive techniques such as dissemination of the guideline in print or electronic form: this approach alone is unlikely to result in changes in health professional practice or the desired health outcomes [9]. Although educational outreach [2], multifaceted approaches [22], reminders [23] and audit and feedback [14] are active techniques that are effective in low-income countries, reminders and multifaceted approaches were almost never mentioned in the WHO guidelines.

There are several possible reasons why techniques shown to be effective were not included in WHO guidelines. A ‘multifaceted approach’ is hard to define and, thus, may be difficult to reproduce. These approaches are also complex and can be costly to scale up. ‘Reminders’ (especially computerised) could also be difficult to implement in countries where resources are extremely limited. Yet, the proven effectiveness of these techniques should nonetheless be taken into consideration when developing implementation plans. Mentioning these techniques as options in the guideline would also broaden the target audiences’ choices during the implementation phase. By having a range of techniques to choose from, the local policymakers and programme managers can select the most suitable techniques for their local setting, based on evidence.

The prevalence of policy-based techniques, such as implementing new laws, working with local health officials and building supportive consensus among stakeholders are especially relevant for public health guidelines that must be implemented at the level of the health system. Policy implementation techniques make it necessary to contextualise and adapt WHO guidelines, which are global in scope, to local conditions. Yet, simply turning a global guideline into a local standard is only the first step of implementation. Countries with limited resources are faced with the task of considering their health systems and, in some cases, political systems when contextualising and adapting guideline recommendations. Even implementation of a relatively straightforward recommendation, such as a change in recommended malaria treatment, can incur significant costs and health system changes [24]. Few WHO guidelines provided detailed plans for contextualising guidelines and systematically gathering the information required for implementation, such as those provided by the SUPPORT tools [19, 25]. Implementation sections of WHO guidelines should include guidance on contextualising and adapting guideline recommendations, as well as evidence-based implementation strategies.

Active and effective implementation techniques that could be used in local settings should be described in the guideline to facilitate the tasks of local policymakers and programme managers. For example, after WHO guidelines are contextualised and adapted to the local context, active techniques such as paper-based reminders or audit and feedback could be used to facilitate implementation of the recommendations.

Currently, the recommendations in the implementation section of WHO guidelines are given in no particular order. A step-wise process could provide clearer direction for implementing new guidelines. One possible model for the layout of the implementation section would be first, consult stakeholders, complete a process for contextualising the guidelines, adapt the guideline to local settings, disseminate the guideline and ancillary documents, use active implementation techniques and, finally, monitor for the effectiveness of the implementation. Listing the steps required for contextualization, adaptation and implementation in the intended order could better direct the local authorities in implementing a new guideline.

An alternative to expanding and structuring the implementation section of each WHO guideline is to require the technical units involved in each guideline to develop a separate implementation document, such as the workbook developed for contextualising the “Optimizing health worker roles to improve access to key maternal and newborn health interventions through task shifting guideline”. This document could contain not only recommendations for specific evidence-based implementation techniques but also additional information that end-users need to consider before a guideline can be contextualised or adapted. These considerations include.

- Applicability to their setting
- The conditions under which the recommended intervention works best
- Feasibility and resource implications;
- Health indicators for monitoring, i.e. how to evaluate the impact if the recommendation is implemented; and
- Effect on equity across population groups and human rights

The vague and non-descriptive nature of implementation plans was commonly observed throughout the study. One could argue that for active implementation techniques such as “training”, the lack of detail gives the local authorities freedom to plan their own programmes. On the other hand, in low-income countries, the local national governments and health systems may not be able to implement effective training due to lack of resources and specific guidance. Previous studies of successful implementation of health guidelines in low-income countries [26] have included extensive description of training programmes, such as the ‘train the trainers model’.

The discrepancy between the effective evidence-based implementation techniques listed in the EPOC taxonomy [12] and the techniques recommended in WHO guidelines, may be because EPOC’s techniques are focused on clinical and health care systems at a local level, instead of on public health at a national or regional level, which is where WHO guidelines are aimed. For example, *WHO guidelines for indoor air quality: dampness and mould (2008)* provide recommendations for safe indoor air quality [27]. Since this guideline does not directly deal with health professional practice or education, some of the active implementation techniques in the EPOC taxonomy are not applicable. Implementation techniques for this guideline should include ways to change national standards of ventilation in buildings, for example. Further research into the effectiveness of various implementation techniques for public health guidelines could also inform the selection of implementation techniques in the future.

More emphasis should also be put on developing tailored interventions as the local context needs to be taken into consideration when implementing guidelines [28]. The phenomenon of recycling whole implementation sections as observed in our study should be avoided as this suggests that very little thought has been put into the implementation of recommendations during the guideline developmental process. By discouraging the recycling phenomenon, guideline panels would be forced to provide more details specific to each recommendation in their implementation plan.

### Strengths and limitations

A limitation of our study is that the coding tool was based on the EPOC taxonomy, which was derived from research on implementation techniques for clinical and healthcare system interventions and not on interventions focused on public health systems.

The strengths of the current study lie first in the fact that our cohort of guidelines included all guidelines approved by the WHO Guidelines Review Committee since the committee’s formation. This provided a wide range of guidelines and gave insights into the evolution of guideline development processes at WHO over the last 8 years. Second, to our knowledge, no previous studies have examined the implementation techniques recommended by WHO guidelines. Third, by categorising these techniques according to the evidence supporting their effectiveness, we identified weaknesses in the implementation plans in many guidelines and made recommendations for improvements in future guidelines.

## Conclusions

The findings of this study add to the body of knowledge about the implementation techniques suggested in WHO guidelines. Revisions of the *WHO Handbook for Guideline Development* [5] should include specific guidance on contextualising and adapting guidelines, as well as options for active, effective techniques that can be used for implementation of public health guidelines in low-income countries. This can include providing a selection of evidence-based guideline implementation techniques, structuring and increasing the level of detail in the section of the guideline focused on implementation or creating accompanying documents that provide information on what is needed to adapt and implement a guideline. Without the proper implementation of guidelines by their intended users, the financial and human resources expended in the development of WHO guidelines is wasted.

### Availability of data and materials

The raw data of this study will be made available to the publisher. An Excel spreadsheet of the data extraction was included in the submission of the manuscript.

## Abbreviations

EPOC: Cochrane Effective Practice and Organisation of Care Group
GRC: World Health Organisation Guidelines Review Committee
WHO: World Health Organisation
